# Concreting a sustainable future: A dataset of alkali-activated concrete and its properties

**DOI:** 10.1016/j.dib.2023.109525

**Published:** 2023-08-28

**Authors:** Benjamin Moreno Torres, Christoph Völker, Rafia Firdous

**Affiliations:** a Bundesanstalt für Materialforschung und -prüfung, Technische Universität, Berlin, Germany; b Bundesanstalt für Materialforschung und -prüfung, Berlin, Germany; c Technische Universität, Berlin, Germany

**Keywords:** Alkali activated concrete, Compressive strength, Carbon footprint, Fly ash, Ground granulated blast furnace slag

## Abstract

This data article introduces a dataset comprising 1630 alkali-activated concrete (AAC) mixes, compiled from 106 literature sources. The dataset underwent extensive curation to address feature redundancy, transcription errors, and duplicate data, yielding refined data ready for further data-driven science in the field of AAC, where this effort constitutes a novelty. The carbon footprint associated with each material used in the AAC mixes, as well as the corresponding CO_2_ footprint of every mix, were approximated using two published articles. Serving as a foundation for future expansions and rigorous data applications, this dataset enables the characterization of AAC properties through machine learning algorithms or as a benchmark for performance comparison among different formulations. In summary, the dataset provides a resource for researchers focusing on AAC and related materials and offers insights into the environmental benefits of substituting traditional Portland concrete with AAC.

Specifications Table

**Table:** 

Subject	Material Characterization
Specific subject area	Alkali-activated concrete dataset: formulation, material properties, performance metrics.
Type of data	Table
How the data were acquired	The dataset was acquired through a systematic literature review of published studies on alkali-activated concrete (AAC) mixtures by Xi et al. [1] and have been curated and integrated into a singly data set by the authors of this contribution. Data were extracted from experimental results reported in these studies, which were obtained using various instruments and techniques, such as compressive strength tests, flexural strength tests, and material characterization methods. The dataset compilation involved tabular organization of the formulations and their corresponding features. No questionnaires or surveys were used in the data acquisition process.
Data format	Raw
Description of data collection	Data collection involved a systematic literature review of studies on alkali-activated concrete (AAC) mixtures. Formulations and features were extracted from these studies, considering experimental conditions and material properties. Inclusion criteria included relevance, sufficiency, and significance of the studies. Exclusion criteria encompassed interpolated data and inconsistencies. Normalization of data was left to future users.
Data source location	To optimize the space provided in this table, we refer to the raw data in the following link: https://zenodo.org/record/7805018/files/Bibliography.txt?download=1
Data accessibility	Repository name: An Alkali-Activated Concrete Dataset for Sustainable Building Materials (Zenodo) Data identification number: D.O.I.:10.5281/zenodo.7805018 Direct URL to data: https://zenodo.org/record/7805018#.ZDUCiXbP2Ul

## 1 Value of the Data

• The dataset provides a comprehensive and organized compilation of alkali-activated concrete (AAC) formulations and features, serving as a valuable reference for researchers, engineers, and industry professionals.
• The dataset benefits stakeholders in the AAC community, including material scientists, engineers, construction industry professionals, and researchers focusing on sustainable construction materials.
• The data can facilitate the identification of research gaps, serve as input for machine learning algorithms (MLAs) to predict AAC properties, and assist in designing new AAC formulations with enhanced performance.
• Industry professionals can use the dataset as an objective benchmark for comparing the performance of AAC mixtures, thereby promoting consistent advancements in AAC technology.
• The dataset's organization facilitates further analysis and exploration, enabling the development of new experiments, optimization strategies, and environmentally friendly concrete solutions.

As a baseline resource, the dataset has the potential to inspire future revisions and expansions, leading to a continually evolving and up-to-date knowledge base for the AAC community.

## 2 Objective

Concrete is the most used building material globally, and the production of its main component, cement, accounts for approximately 8% of worldwide CO_2_ emissions [2]. In response, the construction industry has pursued sustainable alternatives to building materials to reduce its environmental impact. AAC has emerged as a promising alternative, offering the potential to significantly decrease CO_2_ footprint compared to ordinary Portland concrete (OPC) [3]. To harness the advantages of AAC and facilitate its adoption, it is imperative for the research community to understand the relationships among its components, curing processes, and mechanical properties. Data-driven solutions have become crucial in unlocking AAC's potential and fostering innovative, eco-friendly construction practices [4,5]. Although it is explicitly recognized here that the scope of the present compilation does not encompass the latest advancements in this field, the selection criteria for both the compilation and the process that enhances this data publication were carefully considered. While recognizing the important contributions and advancements in the field as indicated by recent studies, the scope of the present compilation is primarily focused on optimizing data utility for the AAC research community. This involves careful curation, standardization, and presentation of a comprehensive dataset, rather than providing a comparison of our results with the latest research findings. We believe that this clear focus adds value to our publication and promotes reproducibility and reusability of the data.

In compliance with the FAIR initiative^1^, which delineates the optimal conditions for data utilization in scientific research, this article presents a comprehensive dataset on AAC formulations and properties, acting as a resource for both researchers and industry professionals. Prior to its assembly, a meticulous survey was conducted to ascertain the existence of analogous datasets, and the absence thereof substantiated the need for this compilation. The dataset is designed to bridge the gap between experimental observations and practical applications of AAC by offering an interoperable dataset that overcomes challenges posed by the diversity of testing methods and research objectives. It encourages the use of MLA to predict AAC properties, streamlining the design process, optimizing material selection, and facilitating AAC integration into construction projects. The dataset enables AAC formulation comparison and promotes the use of these materials. It also lays the groundwork for future research in the AAC field and fosters collaboration among researchers, material scientists, and industry stakeholders. This study holds importance in its provision of a high-quality, ready-to-use dataset for the AAC research community, requiring minimal manipulation for application in various data-intensive research areas, including but not limited to, algorithm development, machine learning model training, and theoretical validation.

## 3 Data Description

The data used in this work is a compilation of alkali-activated concrete formulations and corresponding compressive strengths from [1] . The underlying data have been integrated into a single table and has been enriched with CO_2_ footprints calculated from CO_2_ values of constituents obtained from the literature. Compressive strength and CO_2_ emissions are critical properties for selecting concrete for various applications. While the dataset primarily focuses on strength measures, it also includes additional properties where available. Several studies in our dataset have investigated other crucial characteristics of alkali-activated concrete such as porosity, abrasion, and workability. These attributes are included wherever possible to enrich the dataset, albeit their representation may not be as extensive as desired. Increased focus on these properties in future research would certainly contribute to a more comprehensive understanding of the material. The data can be accessed in [6].

Table 1 provides an overview of the dataset, including the original references, number of mixes, binder types, shape of specimens, and their age at compression test. The dataset comprises over 1630 materials, each described by common features, corresponding compressive strength values from the laboratory, and the calculated carbon footprint. The features can be grouped into nine categories, presented in the format **(Number of features of the category) Name of the category**: Description of the category:

1- **(3 features) Identification Features**. Idx_Sample corresponds exactly with the same numeration used by [6] and acts as universal identifier for every AAC formulation. Ref. corresponds to the reference number used in this document where the mix is sourced. Should there be any internal identifier for the AAC mix in the reference, it is recorded in the Mixture Code in Ref.
2- **(98 features) Binder Oxides Composition**. Molecular composition of the binder powder given in weight percent, providing information on the chemical reactivity of the constituents. In instances where more than one binder is employed, the weighted ratio is computed for each oxide molecule, and the breakdown for every type of molecule is presented for each binder source.. These breakdowns employ the 13 more common oxides to describe the chemical composition for the binder, together with the Loss on Ignition when available.
3- **(14 features) Binder Structure, Content and Density**. This category covers the specific surface area and specific gravity of the precursors. Weights and density of each type of precursor, namely fly ash (FA), ground granulated blast furnace slag (GGBFS), metakaolin (MK), silica fume (SF), other supplementary cementitious materials (SCM) and ordinary Portland concrete (OPC), are included.
4- **(5 features) Aggregate Amount and Density.** This category includes the weights and density of coarse and fine aggregates as well as their quantity per cubic meter of concrete. The Total aggregates (kg in 1m3 mix) feature, although redundant, is preserved to avoid an extra step in case this feature is of interest.
5- **(13 features) Alkali Activator Content and Concentration**. Across all reference sources, the activator is a mix of sodium hydroxide [Na(OH)] solution and sodium silicate [Na_2_SiO_3_] solution. The specific gravity of both, the amount (kg per 1m3 of AAC mix) and chemical percentage composition of the activator constituents are described, together with the molar concentration of the Na(OH) solution.
6- **(3 features) Workability Features**. Amount of additional water and/or superplasticizer indicated. This includes the total amount of water in the concrete mix.
7- **(4 features) Curing Features**. Curing process described quantitatively, with non-quantitative characteristics discarded except for Final curing temperature where values like “Oven”, “Ambient”, “External exposure” or “Sealed outdoor” are preserved.
8- **(3 features) Sample Dimensions**. This category specifies the side length for cubic specimens and height and diameter for cylindrical specimens.
9- **(108 features) Properties of Fresh Mix and AAC Specimens**. This category includes mechanical and structural properties of the cured specimen, as well as workability, porosity measures and setting time of fresh mix.
10- **(1 feature) CO_2_ footprint**. The carbon dioxide (CO_2_) footprint for each mixture has been quantified using the methodology outlined in [7] and further augmented by integrating a linear regression analysis of the CO_2_ footprint attributed to oven curing, as derived from [3].

**Table 1:** Brief summary of the number and type of AAC mixes provided by each reference.

## 4 Experimental Design, Materials and Methods

**Data validation and curation**. The methodology employed in the data validation and curation process involved a carefully executed workflow that ensured the creation of a comprehensive, accurate, and user-friendly dataset for further research and analysis in the field of AAC. Several methodological aspects were adopted from the original compilation by [1], including the selection of relevant papers and the time period determining that selection. This work enhances the approach of [1] by refining the dataset and addressing any inconsistencies, thereby amplifying its utility and value for the research community. The process aimed to develop a unified approach for assessing influential factors on the hardened properties of AAC in an assigned curing regime, using both sufficiency (quantity) and significance (quality) as selection factors for choosing pertinent AAC studies.

The process began with the elimination of redundant features such as columns representing equivalent information, simplifying the dataset and discarding unnecessary data points. Fields facilitating future use, such as "Total Aggregate" and complementary percentages, were preserved to ensure that the dataset remains user-friendly and informative.

Outliers were subsequently identified and carefully examined in the original reference source, with potential transcription errors corrected to maintain accuracy throughout the dataset. In cases where multiple binders were used, the molecular compositions were reviewed, and the weighted sum was calculated to provide a comprehensive understanding of the binder's impact on the AAC properties.

As the process unfolded, repeated mixtures appearing in different studies were detected, and duplicate entries removed to avoid redundancy. This step was essential in preserving the dataset's integrity and ensuring the uniqueness and value of the information provided.

The dataset's accuracy was further improved by inspecting specimen dimensions and the timing of compression tests, ensuring data consistency and reliability. Additionally, all unit consistency was verified, ensuring uniformity across the dataset and facilitating ease of use for future researchers and industry professionals.

Finally, mixtures resulting from numerical interpolations were excluded from the dataset, as they may not accurately represent real-world applications and could potentially skew the dataset's overall relevance and applicability.

By executing this extensive workflow as a fluent process, the data validation and curation methodology resulted in a dataset that is not only comprehensive and accurate but also provides valuable insights and information for the ongoing research and development efforts in the field of AAC.

For the calculation of the carbon footprint of each AAC mixture, the footprint associated with both the production/extraction of the constituent materials and the oven curing of some mixtures has been considered. Both factors are summarized according to the following heuristic equation:

$$CO_{2}\;emissions\;\left(\frac{t}{m^{3}}\right)=\sum_{i}w_{i}\cdot m_{i}+\left(0.6417\cdot T-16.0417\right)\cdot t$$

*where:*

w_i_ CO_2_ emissions (t) to produce 1 ton of the mix component i
m_i_ Mass of a mix component i in t/m3 of fresh mix
T    Heat curing temperature if higher than 25°C; otherwise, t is set to zero
t    Curing time expressed in days

The first part of the equation sums the carbon footprint **w_i_** weighted with the mass of the individual precursor materials **m_i_** . [7] provide the values shown in Table 2.

**Table 2:** CO_2_ footprint for constituent materials in (tones of CO2)/(tones of constituent material).

	OPC	Fly Ash	GGBFS	Silica Fume	Metakaolin	Aggregates	Superpla- sticizer	NaOH Dry	Sodium silicate solution	Sodium silicate dry
*w (t/t)*	0.84	0.004	0.052	0.014	0.33	0.0048	1.88	1.915	0.36	1.222

**Limitations.** The compilation, which covers a limited time period, is not exhaustive and might inadvertently omit relevant studies. The publication of this dataset is expected to prompt its review and augmentation by the research community.

Representing the curing process of alkali-activated concrete mixtures in a tabular form is challenging due to the absence of a universally accepted sequence of curing steps and the complexity of tabular representation. The approach adopted in this dataset is streamlined, concentrating on temperature and duration as the principal factors affecting curing, but it might entail notable omissions. While the detailed descriptions of the curing process in most studies facilitate repeatability, they also accentuate the significant variation in the presentation of curing procedures, which is critical for ensuring the performance attributes of the construction material. Consequently, certain specific elements of the curing process, such as the resting phase prior oven inclusion or the conditions during the resting phase, may not be comprehensively represented in the dataset.

**Fig. 1:**
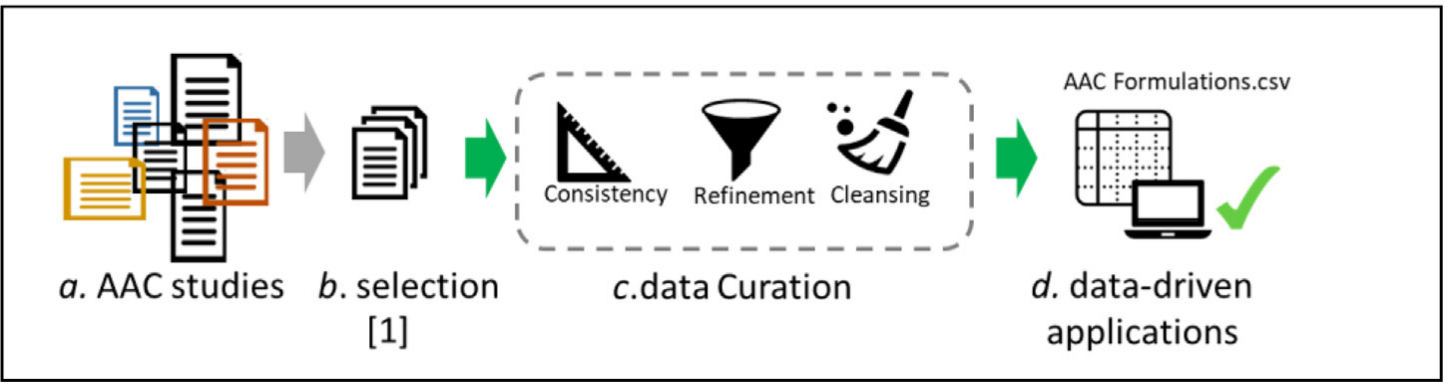
Graphical Abstract. From left to right: **a.** represents the set of research papers that study the different features of AAC. [1] Selected in **b.** a subset of these papers that served their research goals. The interest of this compilation in the AAC community made the **c.** data curation process worth the effort. The format of the data is ready for the **d.** data-driven applications.

**Fig. 2:**
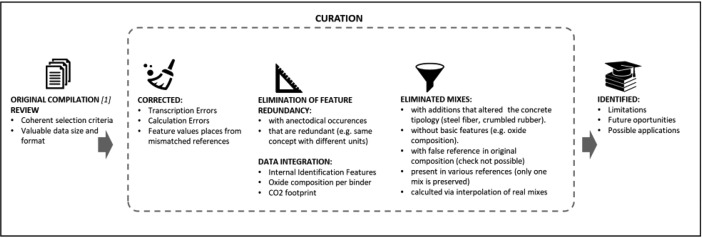
Flowchart. From left to right: The original compilation has desirable characteristics but is not directly re-usable. Into the curation concept we include, from a general overview, the steps present in the area of the figure marked as “curation”. This process allowed the identification of the limitations of the curation process (in terms of time and resources and final degree of reliability), the opportunities that the publication of the data can provide in terms of the inclusion of new mixes, and also the possible application of the data into data analysis / machine learning workflows.

The dataset incorporates estimated carbon footprint calculations for each specimen drawing on existing sources [7] and [2]. Nonetheless, this simplified model does not consider certain factors, such as the influence of material transportation or the disparities between mass production and controlled laboratory production, due to the lack of pertinent data. Future research could concentrate on refining the modeling and computation of these factors, along with other environmentally significant aspects concerning the features of each mixture, to yield a more holistic understanding of the environmental ramifications (Fig. 1, Fig. 2)

**Data usage.** This dataset is a crucial element in the effort to create a fundamental benchmark for use by diverse stakeholders within the AAC community. In material characterization, the dataset serves two main purposes: facilitating the identification of research gaps and supplying input for algorithms dedicated to developing predictive models. For the industrial sector, the dataset acts as an objective standard for comparing the performance of AAC concretes, thereby encouraging consistent progress across diverse domains.

The dataset ensures uniformity in the presentation of each feature by adhering to consistent units of measurement. However, it is recommended that future users of this dataset normalize the features to mitigate any bias in their significance when employed in predictive models. Within concrete production, kilograms per cubic meter is the prevalent unit for denoting material quantities in mix formulations, and this dataset conforms to this practice while utilizing the international metric system for all other units. The dataset is primarily an accumulation, so not all features are present for every formulation. To achieve internal consistency, users of the dataset need to conduct a filtering and selection process according to their specific needs.

## Ethics Statements

Not applicable.

## Statement

During the preparation of this work the author(s) used ChatGPT in order to improve the English grammar and avoid spelling mistakes. After using this tool/service, the author(s) reviewed and edited the content as needed and take(s) full responsibility for the content of the publication.

## CRediT authorship contribution statement

**Benjamin Moreno Torres:** Methodology, Validation, Data curation, Writing – original draft, Writing – review & editing. **Christoph Völker:** Conceptualization, Writing – review & editing, Supervision, Project administration. **Rafia Firdous:** Data curation, Writing – review & editing.

## Data Availability

An Alkali-Activated Concrete Dataset for Sustainable Building Materials (Original data) (Zenodo).

## Footnotes

1 https://www.go-fair.org/fair-principles/.

## References

[1] Xie T., Visintin P., Zhao X., Gravina R. (2020). Mix design and mechanical properties of geopolymer and alkali activated concrete: review of the state-of-the-art and the development of a new unified approach. Construct. Build. Mater..

[2] Andrew R.M. (2018). Global CO_2_ emissions from cement production. Earth Syst. Sci. Data.

[3] Yang K.-H., Song J.-K., Song K.-I. (2013). Assessment of CO2 reduction of alkali-activated concrete. J. Clean. Prod..

[4] Li Z. (2022). Machine learning in concrete science: applications, challenges, and best practices. npj Comput. Mater..

[5] C. Völker et al., *Green building materials: a new frontier in data-driven sustainable concrete design*. 2023. doi:10.13140/RG.2.2.29079.85925.

[6] Torres B.M., Völker C., Firdous R. (2023). An alkali-activated concrete dataset for sustainable building materials. Zenodo.

[7] Alsalman A., Assi L.N., Kareem R.S., Carter K., Ziehl P. (2021). Energy and CO_2_ emission assessments of alkali-activated concrete and ordinary Portland cement concrete: a comparative analysis of different grades of concrete. Clean. Environ. Syst..

